# Acute Appendicitis With Periappendiceal Abscess Caused by Migration of an Endoscopic Clip Into the Appendix: A Case Report

**DOI:** 10.7759/cureus.105939

**Published:** 2026-03-26

**Authors:** Risako Ono, Tatsuya Yamada, Tetsuya Danbara, Naoki Hashimoto, Hirofumi Tsutsumi

**Affiliations:** 1 Department of Gastroenterological Surgery, Tatebayashi Kosei General Hospital, Tatebayashi, JPN

**Keywords:** acute appendicitis, appendectomy, endoscopic clip, foreign body, retained appendicolith

## Abstract

Acute appendicitis is a common surgical emergency, most often caused by luminal obstruction. However, appendicitis resulting from the migration of an endoscopic clip is extremely rare. Because non-absorbable foreign bodies are unlikely to be expelled spontaneously from the appendix, their presence has important implications for management. An 82-year-old woman presented with right lower abdominal pain. Four years earlier, she had undergone colonoscopic polypectomy with endoscopic clipping for hemostasis. Contrast-enhanced computed tomography revealed a periappendiceal abscess and a high-density foreign body near the appendiceal base, suggestive of a migrated endoscopic clip. Initial management with percutaneous abscess drainage and intravenous antibiotics was performed according to institutional protocol; however, the patient’s condition deteriorated, with worsening inflammatory markers and signs of peritonitis. Emergency surgery was therefore undertaken on hospital day 2. Open appendectomy with partial cecectomy and intraperitoneal drainage was performed. Examination of the resected specimen revealed an endoscopic clip within the appendiceal lumen. Histopathological analysis confirmed phlegmonous acute appendicitis. Appendicitis caused by non-absorbable foreign bodies, such as endoscopic clips, is unlikely to resolve with conservative management. Based on this case and a review of the literature, early surgical intervention may be considered, even in the presence of an abscess. Recognition of this condition is essential to avoid delays in definitive treatment.

## Introduction

Acute appendicitis is the most common indication for emergency abdominal surgery worldwide [1]. The etiology of acute appendicitis remains incompletely understood [2]; however, luminal obstruction is widely recognized as a key etiological factor, and the underlying causes of obstruction are heterogeneous [1,3]. Reported foreign bodies include dental materials, fish bones, and needles [4]. Among these, foreign body-induced appendicitis represents a distinct but uncommon clinical entity, accounting for approximately 3% of cases [5,6]. However, obstruction caused by an endoscopic clip as a foreign body has rarely been reported and is considered extremely uncommon [4]. To date, only a limited number of cases have been reported in the literature, highlighting both the rarity and the limited understanding of its pathophysiology and optimal management.

Herein, we report a rare case of acute appendicitis caused by migration of an endoscopic clip into the appendix and discuss its clinical implications with a focus on pathophysiology and management strategy.

## Case presentation

An 82-year-old woman presented with right lower abdominal pain. She had a past medical history of hypertension and gastroesophageal reflux disease, both diagnosed at 50 years of age. Her surgical history included total hysterectomy with bilateral oophorectomy at 42 years, cholecystectomy at 59 years, and total knee arthroplasty at 72 and 77 years.

**Figure 1:**
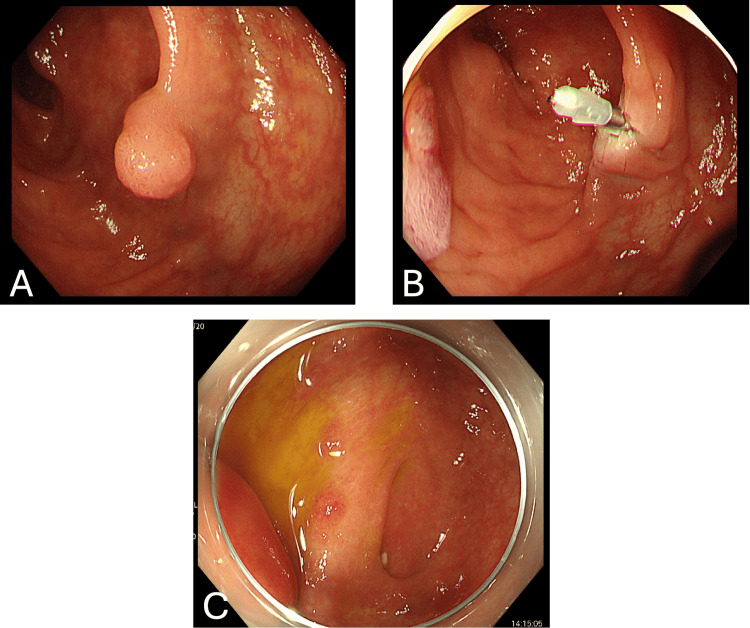
Colonoscopic findings prior to presentation (A, B) Colonoscopic images obtained four years before the onset of appendicitis. A polyp located in the ascending colon was removed by endoscopic polypectomy, and hemostasis was achieved using an endoscopic clip. (C) Follow-up colonoscopy performed one year before admission showed no retained endoscopic clips in the colonic lumen, and no abnormalities were observed at the appendiceal orifice.

Four years prior to admission, the patient underwent three polypectomies for polyps in the cecum and ascending colon. Hemostasis was achieved using an endoscopic clip at one of the sites (Figure 1A, 1B). One year prior to admission, follow-up colonoscopy after polypectomy revealed no retained endoscopic clips in the colonic lumen, and no abnormalities were observed at the appendiceal orifice (Figure 1C).

Three days before the presentation, she developed lower abdominal pain and visited a local clinic, where oral levofloxacin was prescribed. Because the symptoms were severe, she visited another clinic the same day. Right lower abdominal pain, tenderness, and rebound tenderness were noted; however, the treatment plan remained unchanged. Two days later, as the symptoms and physical findings had not improved, she was referred to our department.

On examination at presentation, the body temperature was 36.0 °C, blood pressure was 129/62 mmHg, pulse rate was 67 beats/minute, and oxygen saturation (SpO₂) was 98% on room air. Tenderness and rebound tenderness were present in the right lower abdomen, while muscular guarding was absent. Laboratory tests showed a white blood cell count of 13,500/mm³ and a C-reactive protein level of 17.47 mg/dL, indicating significant inflammatory elevation.

**Figure 2:**
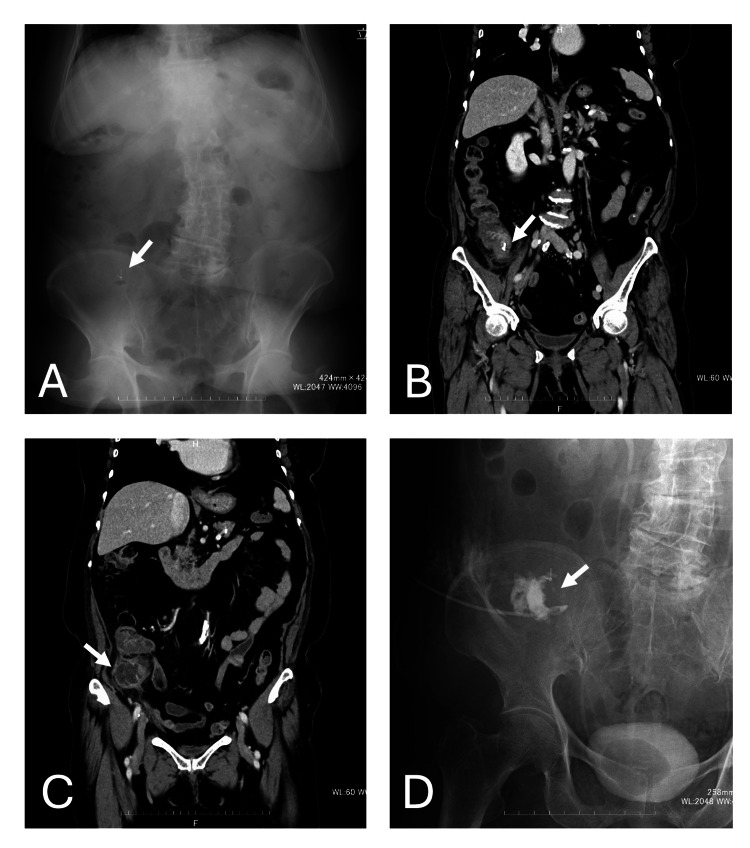
Imaging findings at admission and percutaneous abscess drainage. (A) Abdominal radiograph demonstrating a metallic foreign body (arrow) in the right lower abdomen, suspected to be an endoscopic clip. (B, C) Contrast-enhanced computed tomography showing a periappendiceal abscess adjacent to the cecum and a high-density foreign body near the base of the appendix (arrow), consistent with an endoscopic clip. (D) Percutaneous abscess drainage performed under ultrasound and fluoroscopic guidance. A 7-Fr pigtail catheter (arrow) was placed within the abscess cavity.

Abdominal X-ray showed a foreign body, suspected to be an endoscopic clip, in the right lower abdomen (Figure 2A). Contrast-enhanced computed tomography (CECT) showed a 45-mm abscess cavity on the right ventral side of the cecum. The normal appendix was not visualized, and acute appendicitis with a periappendiceal abscess was suspected. Additionally, a high-density structure was observed near the appendiceal base, which strongly suggested appendiceal inflammation associated with a foreign body (Figures 2B, 2C).

Based on the patient’s clinical presentation and imaging findings, several differential diagnoses were considered, including acute appendicitis with a periappendiceal abscess, cecal diverticulitis, and localized perforation associated with an appendicolith. The anatomical localization of the lesion and the absence of findings suggestive of diverticulitis or generalized perforation further supported the diagnosis of foreign body-induced appendicitis.

Management with percutaneous drainage and antibiotics was selected in accordance with institutional practice for abscess-forming appendicitis. This approach was considered appropriate for achieving initial infection control while avoiding immediate surgery in an inflamed operative field. Percutaneous abscess drainage was performed on hospital day 1 under local anesthesia using real-time ultrasound guidance and fluoroscopy. A 7-Fr pigtail catheter was placed within the abscess cavity (Figure 2D).

Post-procedural CT confirmed appropriate catheter placement within the abscess cavity and showed no injury to adjacent organs. Conservative management was initiated, including bowel rest with fasting, intravenous fluid support, and intravenous antibiotics (cefmetazole 2 g/day). 

**Figure 3:**
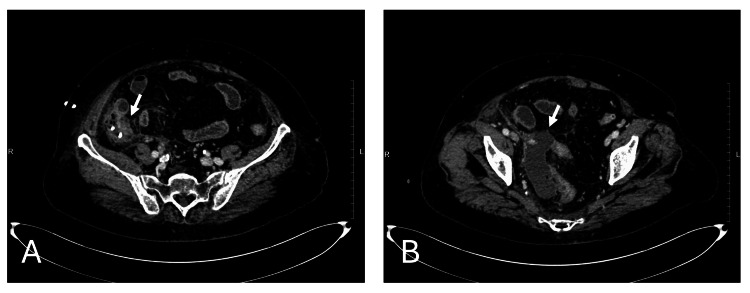
Contrast-enhanced CT findings on hospital day 2. (A) The pigtail catheter remained within the abscess cavity; however, the abscess had not decreased in size. A high-density foreign body suspected to be an endoscopic clip was still present within the abscess cavity (arrow). (B) Increased pelvic ascites and newly developed peritoneal thickening (arrow) suggested worsening peritonitis.

On hospital day 2, abdominal distension worsened. Laboratory tests demonstrated worsening inflammatory markers, with a white blood cell count of 22,700/mm³ and a C-reactive protein level of 31.82 mg/dL. CECT revealed that although the catheter remained within the abscess cavity, the maximum diameter of the abscess had not decreased, suggesting insufficient drainage. A high-density foreign body suspected to be an endoscopic clip remained within the abscess cavity (Figure 3A). In addition, increased pelvic ascites and newly developed peritoneal thickening suggested worsening peritonitis (Figure 3B). Diffuse small bowel dilatation was also observed, consistent with paralytic ileus secondary to inflammation.

Because conservative management with percutaneous drainage and antibiotics was ineffective, emergency surgery was performed on hospital day 2. 

Given the patient’s history of prior laparotomy and marked abdominal distension due to paralytic ileus, an open surgical approach was selected. Under general anesthesia, a lower midline incision was made. Contaminated ascitic fluid was observed within the peritoneal cavity. After the dissection of inflammatory adhesions in the pouch of Douglas, a large amount of white, purulent fluid was drained.

Upon entering the abscess cavity lateral to the cecum, the previously placed pigtail catheter was identified. Blunt dissection of inflammatory adhesions revealed a markedly enlarged appendix approximately the size of a thumb. After dissection of the mesoappendix and exposure of the appendiceal base, partial disruption of the appendix at its base was identified. The appendiceal base showed severe inflammatory involvement with partial disruption, making safe ligation difficult. Therefore, partial cecectomy was performed to ensure complete resection and secure closure. Partial resection of the cecum with the appendix was performed using a linear stapler. 

After thorough peritoneal lavage with warm saline, a 6.5-mm closed continuous suction drain was placed from the right lower abdomen through the abscess cavity to the pelvic floor. Appendectomy with partial cecectomy, intraperitoneal lavage, and drainage was completed. The operative time was 134 minutes, and the estimated blood loss was 70 mL. 

**Figure 4:**
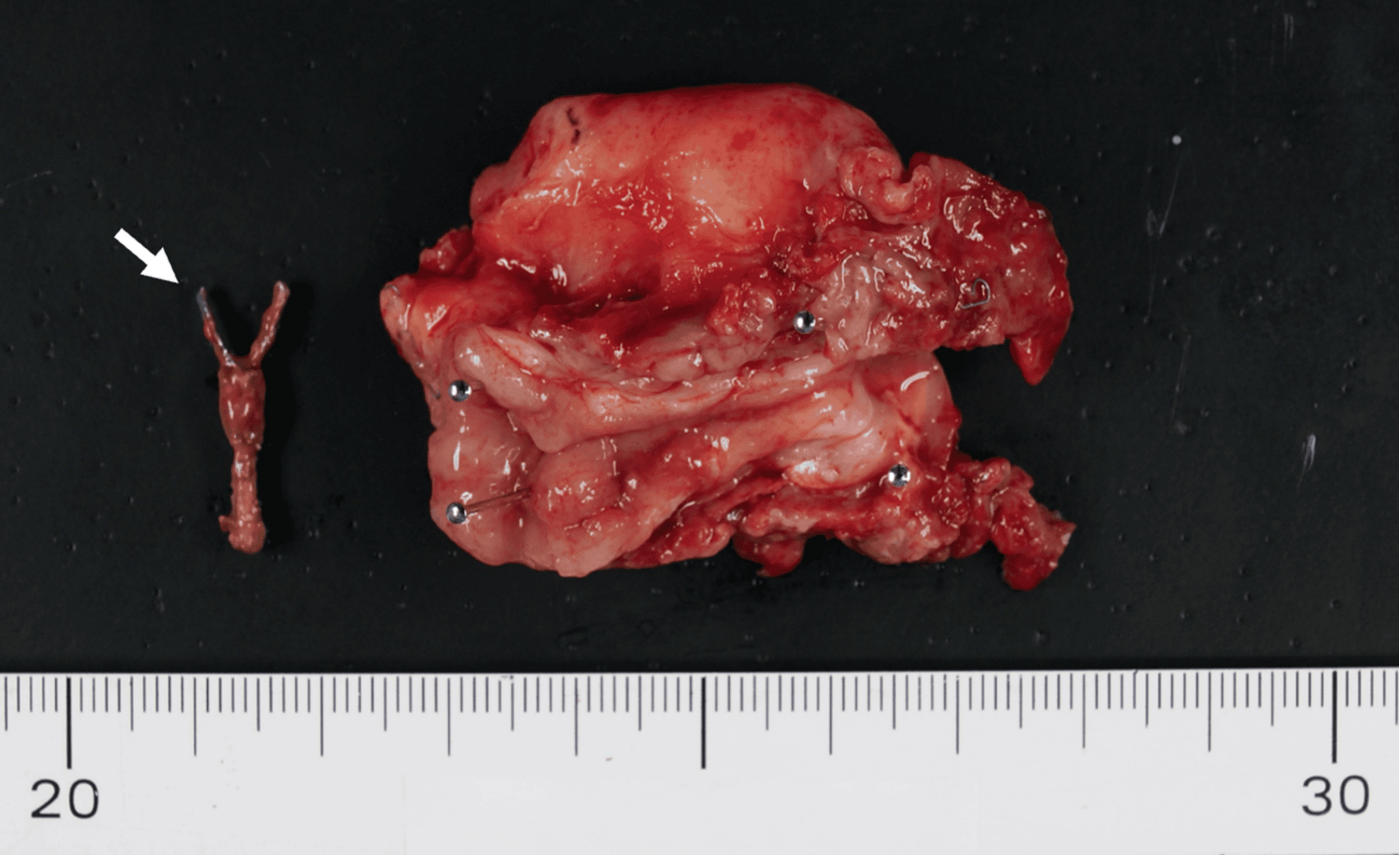
Resected specimen The resected specimen showed marked thickening of the appendiceal wall with disruption at the base. An endoscopic clip was identified within the lumen of the appendix (arrow).

Examination of the resected specimen revealed marked thickening and severe inflammatory changes of the appendiceal wall, disruption at the base, and a migrated endoscopic clip within the lumen (Figure 4). Histopathological examination revealed thickening of the appendiceal wall with disruption at the base. Prominent neutrophilic infiltration, abscess formation, hemorrhage, and congestion were observed, consistent with phlegmonous acute appendicitis. No evidence of neoplasia was identified. The diagnosis of foreign body-induced appendicitis was ultimately confirmed by examination of the resected specimen.

The patient’s paralytic ileus gradually improved during the postoperative period. Oral fluid intake was resumed on postoperative day (POD) 6, and an oral diet was restarted on POD 7. No residual intra-abdominal abscess developed. The drain was removed on POD 8, and the patient was discharged in good condition on POD 17. At outpatient follow-up on POD 38, the postoperative course remained uneventful.

## Discussion

The incidence of appendicitis caused by a foreign body has been reported to be approximately 3% [5,6]. Reported causative foreign bodies include a variety of objects such as dental crowns, fish bones, and needles [4].

A literature search of PubMed and the Japanese database Ichu-shi using the keywords “endoscopic clip,” “endoluminal clip,” and “appendicitis” for publications between 1970 and 2025 identified three cases reported internationally and four cases reported in Japan [6-12]. To our knowledge, the present case represents the eighth reported case worldwide, highlighting the rarity of appendicitis associated with endoscopic clips.

A plausible mechanism for clip-induced appendicitis is that after placement, the clip may detach and migrate distally via intestinal peristalsis. Due to anatomical and gravitational factors, it may become lodged near the appendiceal orifice and subsequently enter the appendiceal lumen. The blind-ended structure of the appendix predisposes to retention, leading to obstruction, bacterial overgrowth, and inflammation. In our case, no clip was observed one year after placement, suggesting delayed migration and supporting this hypothesis.

In the present case, acute appendicitis complicated by a periappendiceal abscess was initially managed conservatively with percutaneous abscess drainage and antibiotics. However, no improvement was observed in clinical, laboratory, or imaging findings, and emergency surgery was required on hospital day 2.

Meta-analyses have shown that antibiotic therapy can be a safe alternative to surgery in adults with imaging-confirmed acute appendicitis [13,14]. However, in patients with an appendicolith, analogous to a foreign body causing luminal obstruction, initial antibiotic therapy carries a higher risk of complications, and nearly 50% of such patients eventually require appendectomy within 12 months [13]. A large randomized controlled trial comparing antibiotic therapy with appendectomy also demonstrated that cases with an appendicolith had a higher rate of conversion to surgery and a significantly increased risk of complications such as abscess formation. The comparison with appendicolith-associated appendicitis should be interpreted with caution, as this represents an extrapolation rather than a direct equivalence. Although both conditions involve luminal obstruction, foreign bodies and appendicoliths differ in composition and biological behavior. Nevertheless, this analogy may provide a useful conceptual framework, particularly given that both conditions are characterized by persistent obstruction that may limit the effectiveness of nonoperative management.

In all seven previously reported cases of appendicitis caused by endoscopic clips, a localized intra-abdominal abscess was observed [6-12]. Similarly, a periappendiceal abscess developed in the present case. In all previously reported cases, surgery was performed within five days of hospital admission, including appendectomy, appendectomy with partial cecal resection, or ileocecal resection (Table 2).

**Table 1:** Endoscopic clip–induced appendicitis reported in the literature Summary of previously reported cases of appendicitis caused by migration of an endoscopic clip into the appendix, together with the present case. The table includes patient demographics, purpose of clip application, organ of clip placement, interval from clip placement to symptom onset, surgical procedure, and postoperative course. HD, hospital day; POD, postoperative day; ESD, endoscopic submucosal dissection

Author	Year	Age (years)/sex	Time to Onset	Purpose of Clip Application	Organ	Operation	Procedure	Approach	Discharge	Language of Publication
Kohama et al. [6]	2009	56/M	4 months	Preoperative marking	Stomach	HD 1	Appendectomy	Open	POD 1	Japanese
Hoshino et al. [7]	2010	66/F	7 years	Polypectomy	Colon	HD 1	Appendectomy with partial cecal resection	Open	Not reported	English
Toyota and Sugawara [8]	2013	81/M	2 years	Polypectomy	Colon	HD 1	Appendectomy	Open	POD 14	Japanese
Shimada et al. [9]	2016	70/M	22 months	Polypectomy	Colon	HD 5	Appendectomy	Open	POD 13	Japanese
Stagnetto et al. [10]	2021	65/F	6 weeks	Polypectomy	Colon	Not reported	Appendectomy with partial cecal resection	Not reported (Laparoscopic ?)	Not reported	English
Yomogida et al. [11]	2023	68/M	2 years	ESD	Stomach	HD 1	Appendectomy	Laparoscopic	POD 4	English
Ishikawa et al. [12]	2025	80/M	7 years	Polypectomy	Colon	HD 1	Ileocecal resection	Laparoscopic	POD 11	Japanese
Present Case	2026	82/F	4 years	Polypectomy	Colon	HD 2	Appendectomy with partial cecal resection	Open	POD 17	English

In cases of appendicitis with abscess formation, favorable outcomes following abscess drainage have been reported [14]. However, abscess formation with fecaliths has also been identified as a predictor of failure of nonoperative management in acute appendicitis [15]. Foreign bodies are not expected to undergo spontaneous absorption and are therefore even less likely than fecaliths to be eliminated from the appendix. Accordingly, in cases of acute appendicitis caused by foreign bodies, early appendectomy should be considered rather than nonoperative management, even in the presence of an abscess.

At our institution, appendicitis with abscess formation is typically managed with initial percutaneous drainage followed by interval appendectomy. This approach is supported by previous studies, which suggest that nonoperative management can be an effective strategy in selected patients with complicated appendicitis [14]. Accordingly, initial conservative treatment was selected. However, this approach may not have been appropriate given the presence of a persistent foreign body. Retrospectively, early surgical intervention might have prevented clinical deterioration. These findings highlight the limitations of standard treatment algorithms and underscore the importance of etiology-based decision-making.

The interval between endoscopic clip placement and the onset of appendicitis varies widely, ranging from six weeks to seven years. Among the eight cases in Table 4, four previously reported cases [7,8,11,12] and the present case (62.5%) developed appendicitis more than two years after clip placement, suggesting that the condition can occur long after the initial procedure. When endoscopic clips are incidentally identified within the appendix, prophylactic appendectomy may be considered as a preventive strategy to avoid the development of acute appendicitis.

An important clinical implication of the present case is that the absence of a visible endoscopic clip on surveillance colonoscopy does not exclude subsequent migration into the appendix. In our case, no clip was identified one year after placement; however, a clip was later found within the appendiceal lumen at the time of presentation. This suggests that clip migration may occur in a delayed and dynamic manner, possibly after detachment from the mucosa and gradual intraluminal movement influenced by peristalsis and gravitational forces. Therefore, clinicians should be aware that even when endoscopic clips are no longer visible within the colonic lumen, delayed migration into the appendix remains a potential mechanism of appendicitis. 

Unlike typical causes of appendicitis, such as fecaliths, non-absorbable foreign bodies cannot be expected to resolve spontaneously and may continue to act as a persistent source of luminal obstruction and inflammation. Therefore, when a non-absorbable foreign body is identified within the appendix on imaging, early surgical intervention may be considered a reasonable approach, irrespective of standard nonoperative management strategies. In retrospect, early surgical intervention may have been a reasonable option in this case, as it could have prevented clinical deterioration, including worsening inflammation and peritonitis. 

This case highlights the importance of tailoring treatment decisions based not only on disease severity but also on the underlying etiology.

## Conclusions

Acute appendicitis caused by foreign bodies is rare but clinically important. In the present case, conservative management with drainage and antibiotics was unsuccessful, necessitating emergency surgery. Similarly, previously reported cases have demonstrated a limited response to nonoperative management, with most patients ultimately requiring surgical intervention. Taken together, these observations suggest that early surgical intervention may be considered as a reasonable option in cases of appendicitis associated with a non-absorbable foreign body. However, given the limited number of reported cases, these findings should be interpreted cautiously, and management decisions should be individualized. In addition, when an endoscopic clip is incidentally identified within the appendix, prophylactic appendectomy may be considered as a preventive strategy. However, this approach should be individualized based on a careful risk-benefit assessment.

## References

[1] Baird DL, Simillis C, Kontovounisios C, Rasheed S, Tekkis PP (2017). Acute appendicitis. BMJ.

[2] Packard E, Groff A, Shahid Z, Sahu N, Jain R (2019). A 'bit' of appendicitis: a case of a foreign object in the adult appendix. Cureus.

[3] Snyder MJ, Guthrie M, Cagle S (2018). Acute appendicitis: efficient diagnosis and management. Am Fam Physician.

[4] Elmansi Abdalla HE, Nour HM, Qasim M, Magsi AM, Sajid MS (2023). Appendiceal foreign bodies in adults: a systematic review of case reports. Cureus.

[5] CO DC (1963). 71,000 human appendix specimens. A final report, summarizing forty years' study. Am J Proctol.

[6] Kohama K, Hiura Y, Hirao M (2009). A case of acute appendicitis caused by marking clip of preoperative gastric cancer after barium enema study.(Article in Japanese). JJAAM.

[7] Hoshino I, Sugamoto Y, Fukunaga T (2010). Appendicitis caused by an endoluminal clip. Am J Gastroenterol.

[8] Toyota K, Sugawara Y (2013). A case of perforation of the appendix caused by aberrantly positioned clips used for endoscopic therapy (Article in Japanese). JJAAM.

[9] Shimada M, Hidaka E, Takayanagi D (2016). A case of acute appendicitis caused by a clip used for endoscopic therapy (Article in Japanese). JJAAM.

[10] Stagnetto M, Coulier B, Pierard F (2021). Colonic hemostatic clip causing perforated acute appendicitis. J Belg Soc Radiol.

[11] Yomogida D, Fujisawa Y, Takeji A (2023). Endoscopic clip-induced acute appendicitis in a patient on chronic hemodialysis: a case report with literature review. Ren Replace Ther.

[12] Ishikawa S, Taki K, Akahoshi S (2025). A case of acute appendicitis due to perforation caused by an endoscopic clip migrating into the appendix after ascending colon polypectomy (Article in Japanese). Jpn J Gastroenterol Surg.

[13] Flum DR, Davidson GH, Monsell SE (2020). A randomized trial comparing antibiotics with appendectomy for appendicitis. N Engl J Med.

[14] Simillis C, Symeonides P, Shorthouse AJ, Tekkis PP (2010). A meta-analysis comparing conservative treatment versus acute appendectomy for complicated appendicitis (abscess or phlegmon). Surgery.

[15] Wakasa Y, Toyoki Y, Kameyama Y (2023). Predictors of nonoperative management failure and recurrence in adults with acute appendicitis: a single-center retrospective study. Surg Gastroenterol Oncol.

